# Behavioral, metabolic, and biochemical alterations caused by an acute stress event in a zebrafish larvae model

**DOI:** 10.1007/s10695-024-01421-7

**Published:** 2024-12-14

**Authors:** Raquel S. F. Vieira, Carlos A. S. Venâncio, Luís M. Félix

**Affiliations:** 1 Centre for the Research and Technology of Agro-Environment and Biological Sciences (CITAB), University of Trás-Os-Montes and Alto Douro (UTAD), Vila Real, Portugal; 2 Inov4Agro, Institute for Innovation, Capacity Building and Sustainability of Agri-Food Production, University of Trás-Os-Montes and Alto Douro (UTAD), Vila Real, Portugal; 3 Department of Animal Science, School of Agrarian and Veterinary Sciences (ECAV), University of Trás-Os-Montes and Alto Douro (UTAD), Vila Real, Portugal; 4 Animal and Veterinary Research Centre (CECAV), University of Trás-Os-Montes and Alto Douro (UTAD), Vila Real, Portugal

**Keywords:** Zebrafish larvae model, Stress response, Welfare markers, Animal welfare, Metabolic rate

## Abstract

Animal welfare is a growing concern in aquaculture practices. Stress induced by handling or transportation can lead to negative impacts on the sector. Zebrafish has raised as an important aquaculture model, but still with few focus on its stress response in early life stages. Therefore, the objective of this study was to improve the evaluation of different markers of the stress response after a stress event in a zebrafish larvae model. Zebrafish larvae (96 hpf) were vortex-stimulated for 1 min at 200 rpm for acute stress induction. After 10 min, 1- and 4-h behavioral larvae outcomes and larvae were sampled to the following quantification: levels of cortisol, lactate, glucose and biochemical biomarkers (reactive oxygen species, superoxide dismutase, catalase, glutathione peroxidase, lipidic oxidation level and protein carbonylation, glutathione s-transferase, acetylcholinesterase, lactate dehydrogenase and ATPase), and the metabolic rate. The cortisol, glucose, and lactate levels had no alterations. At the behavioral level, an increase in the distance swam and in the speed was observed and the metabolic rate also increased according to the behavioral outcomes. The ATPase and GST activity showed a decrease in their activity, probably through osmoregulation changes related to the hypothetic adrenocorticotropic hormone downregulation. Overall, the acute vortex stimulation at low speed induced an early stress response independent of the HPI-cortisol pathway. In addition, this study shows zebrafish early life stages as a sensitive model to acute vortex stimulation, identifying altered parameters which can be used in future work to assess the effect on animal welfare in similar acute situations.

**Graphical Abstract:**

Vortex event of 96 hpf zebrafish larvae model. Stress event was performed with a vortex flow stimulation, 96 h post-fertilization (hpf) larvae (Faught and Vijayan 2018; Castillo-Ramírez et al. 2019). To induce the stress response, larvae were placed on a shaker and subjected to vortex 250 rpm for 1 min. Animals were collected at three different time points (10 min, 1 and 4 h) following the stress-inducing event for sample processing to obtain data using various techniques. Schematic representation of short-term response and long-term response with the data that can be altered corresponding to work data.

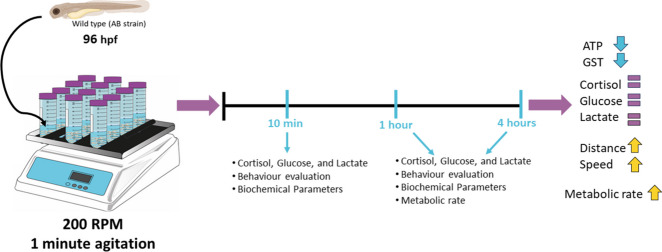

**Supplementary information:**

The online version contains supplementary material available at 10.1007/s10695-024-01421-7.

## Introduction

Over the past years, animal welfare concerns have evolved based on sound scientific knowledge. This has been particularly important in large-scale animals’ production systems such as aquaculture (Martos-Sitcha et al. 2020). With the intensification of aquaculture and the increase concern for animal welfare, there is a need to identify ways to improve and reduce the stress induced by certain practices, such as handling or transportation (Toni et al. 2019). Stress can be defined as a disturbance to homeostasis, contributing to a neurological disorder including several diseases (Chang et al. 2021). Its consequences on neurological and psychological pathways can have negative impacts in the sector. However, few studies have focused on the stress response (Ribas and Piferrer 2014). This is of upmost importance in early life stages as changes in the developmental trajectory can lead to long lasting alterations (Eachus et al. 2021; Swaminathan et al. 2023), despite the plasticity of the developing brain to prevent them (Nagpal et al. 2019; Eachus et al. 2021; Lim and Bernie^2023^). Therefore, a thorough investigation to mechanistically understand the effects that stressful situations can have in the earliest stages of animals is required.

The rodent model has been extensively employed in this research domain (Atrooz et al. 2021, Pesarico, Chagas et al. 2021); nevertheless, specific inquiries regarding comprehensive brain structural and functional alterations triggered by early life stress have posed experimental difficulties. In the last few years, scientists have been using non-mammalian vertebrate as model systems to alternative approaches to gain insights into these aspects (Øverli et al. 2007; Doke and Dhawale 2015, Maximino, Silva et al. 2015). An increasingly used animal model representing aquatic organisms, particularly the teleost family, is the zebrafish. This model exhibits biological characteristics that are representative of a broader range of aquatic species (Ribas and Piferrer 2014) and has been used to assess various neurological, behavioral, and metabolic pathways (Zon and Peterson 2005; McGrath and Li 2008; Mu et al. 2013; Felix et al. 2019; Vieira et al. 2020). In addition, due to these characteristics, it has been considered a promising model for aquaculture research (Varga 2011; Ribas and Piferrer 2014; Ulloa et al. 2014, Lee‐Estevez, Figueroa et al. 2018, Jørgensen 2020), and for nutrition (Ulloa et al. 2014) and diseases (Lee‐Estevez, Figueroa et al. 2018, Jørgensen 2020) studies. It is worth noting that 96 h post-fertilization (hpf), zebrafish larvae are more sensitive than older stages to stressful events (Steenbergen et al. 2011), although presenting a developed nervous system that react to external stimuli (Dai et al. 2014). Overall, this has been described as an acceptable time point for stress evaluation (Alsop and Vijayan 2009; Faught and Vijayan 2018; Castillo-Ramirez et al. 2019). Therefore, the use of zebrafish larvae as a model to study the stress response can optimize aquaculture practices such as handling techniques and transportation, offering insights to enhance fish welfare in larger commercial species in aquaculture.

Yet, there is no standardized method to stimulate the hypothalamo–pituitary–interrenal (HPI) axis, responsible for governing the stress response, in zebrafish early stages. In this context, some acute stressors have been described (e.g., predators, shocks, movement restriction, vortex flow) (Sutanto and De Kloet 1994; Campos et al. 2013; Chang et al. 2021), being the vortex flow stimulation described as an activator of the HPI axis (Chang et al. 2021). However, its relationship with behavior, metabolic rate, and biochemical changes in animals in early stages of development is still to be explored. Therefore, the objective of this work was to deepen and improve parameters evaluation related to stress response in this model that can be applied in different paradigms. Monitoring the effects of stress in these phases can contribute to sustainable management, optimizing the welfare and maintenance of species of economic interest.

## Materials and methods

### Chemicals

MS-222 (ethyl 3-aminobenzoate methanesulfonate, 98%, CAS: 886–86-2) was purchased from Merck (Algés, Portugal). A stock solution (1500 mg L^−1^) was prepared in water and neutralized at pH 7.2–7.4 with sodium bicarbonate. MS-222 was stored at 4 °C until further dilution. A 60X stock solution of E3 medium (5 mM NaCl, 0.17 mM KCl, 0.33 mM CaCl_2_, and 0.33 mM MgCl_2_, pH 7.2) was prepared (Meyers 2018), autoclaved, and freshly prepared at 1X concentration. MS-222 and E3 solutions were stored at 4 °C until further dilution. E3 was diluted to 1X before use, maintained at aquarium temperature and oxygen saturated for 10 min before use. Protease inhibitor cocktail (Cat. No. HY-K0010) was acquired from MedChemExpress (NJ, USA) and stored at − 20 °C.

### Zebrafish maintenance

The zebrafish AB strain was maintained at the University of Trás-os-Montes and Alto Douro in Portugal. Animals were housed in an open water system, aerated, dechlorinated, charcoal-filtered, and UV-sterilized tap water as described earlier (Gomes et al. 2023). The physiochemical properties of City of Vila Real tap water in the tanks were recorded regularly (dissolved oxygen, 7.3 ± 0.7 mg/L; pH, 7.1 ± 0.3; temperature, 28.3 ± 0.4 °C; conductivity, 720 ± 240 μS/cm; alkalinity, 30.1 ± 12.6 mg/L as CaCO_3_; total hardness, 34.0 ± 9.4 mg/L as CaCO_3_; total ammonia nitrogen, 0.5 ± 0.2 mg/L; unionized ammonia, 0.0 ± 0.0 mg/L; nitrite, 0.1 ± 0.1 mg/L; and nitrate, 6.3 ± 0.6 mg/L). The bioterium had a 14:10 light–dark cycle, and the zebrafish were fed twice daily with a commercial diet (Zebrafeed, Sparos Lda., Portugal). Adult zebrafish from AB strain were kept at a maximum density of 40 animals in 20-L glass aquaria (Felix et al. 2017). Reproduction involved pairing male and female zebrafish (2:1 ratio), selected randomly, in small boxes overnight, following a previously described method (Lanzarin et al. 2019; Vieira, Venancio et al. 2021). Collected fertilized eggs were subjected to a Chloramine-T solution (0.5% w/v) for bleaching (Teixido et al. 2019), washed twice with E3 and randomly transferred to 6 well-plates containing E3 medium following established protocols (Westerfield 2007;Varga 2011). The survival rate of embryos until assay was at least 80%, as mentioned in another group work (Gomes et al. 2023). The laboratory experiments involving animals adhered rigorously to ethical principles and complied with both the EU directive (2010/63/EU) and national regulations for animal experimentation and welfare (specifically, Decreto-Lei 113/2013).

### Vortex flow stimulation

On the evening before the experiment, 30 zebrafish larvae with 72 hpf (3 dpf) were transferred to 50-mL centrifugal tubes containing a final volume of 20 mL of E3 medium. To assess the impact of the vortex flow stimulation, 96 h post-fertilization (hpf) larvae were used according to the work carried out by (Faught and Vijayan 2018 and Castillo-Ramírez et al. 2019). The first reference showed that stress caused by a vortex is visible effects on animals after 1 min, this article was adapted the method described previously in which in just 30 s it was possible to verify changes in the animals (Lowe and Wells 1996; Stouthart et al. 1998; Alsop and Vijayan 2008; Faught and Vijayan 2022). To induce the stress response, larvae were placed on a shaker and subjected to vortex stimulation (200 rpm) for 1 min. Animals were collected at three different time points (10 min, 1 and 4 h) following the stress-inducing event.

### Cortisol, glucose, and lactate quantification

For cortisol quantification, five replicates of 30 larvae (*n* = 5) were collected following the stress-inducing event. Briefly, animals were euthanized using an overdose of MS-222 (0.4 g L^−1^) and transferred to microtubes with 100 µL of PBS and protease inhibitor cocktail (1: 100, v:v). A control group of unstressed larvae, also placed in centrifugal tubes and subjected to equal handling conditions and temperature, was euthanized immediately before stress event using an overdose of MS-222 (0.4 g L^−1^) complemented with an excess of 1% sodium hypochlorite for 5 min (Health 2009, Strykowski and Schech 2015).

To assess cortisol levels, samples were treated as described before (Vieira et al. in press). After homogenization in a Tissuelyser II during 90 s at 30 Hz (Qiagen, Germany) and centrifugation of samples at 12,000 g for 10 min at 4 °C in a refrigerated centrifuge (Labnet Prism R, NJ, USA), the supernatant was collected to a new tube to which 1 mL of diethyl ether was added. The samples were incubated on an orbital shaker overnight at room temperature to extract the maximum amount of cortisol (Laberge, Yin-Liao et al. 2019). Then, after extraction proceed, the samples were placed at − 20 °C for 1 h, and the organic phase collected. The diethyl ether was evaporated in a centrifugal vacuum concentrator (Labconco CentriVap 78,120–02 Mobile Centrifugal Concentrator System) and, after evaporation, 200 µL of PBS was added. Samples were left overnight at 4 °C before cortisol quantification proceeded according to the Cortisol Enzyme Immunoassay Kit instruction manual provided (Arbor Assaystm DectectX®, Ann Arbor, USA). The plate was then read by optical density at 450 nm.

For glucose and lactate quantification, new samples were collected, homogenized as cortisol samples and centrifuged at 13,000 rpm for 10 min at 4 °C in a refrigerated centrifuge (Labnet Prism R, NJ, USA) (Brun, van Hage et al. 2019). The glucose kit (ref. 1,001,200, Spinreact, Barcelona, Spain) and the lactate kit (ref. 1,001,330, Spinreact, Barcelona, Spain) were used per manufacturer instructions using 10 µL of sample. The optical density of the plate was red at 505 nm for lactate and at 340 nm glucose on a PowerWave XS2 microplate scanning spectrophotometer (Bio-Tek Instruments, USA).

### Behavior evaluation

To evaluate behavioral features from the stress event, 96 hpf larvae (*n*= 24) were placed, individually, in a 48-well plate containing E3 medium which was on top of a white background on a mini-LED shadowless light (Puluz, Shenzhen, China) at room temperature (25 ± 1 °C). The procedure for evaluated behavior as being descript by work group (Vieira 2024b). After a 10-min habituation, the video record was started using a digital camera (1920 × 1080 resolution) at a sampling rate of 30 frames per second (fps). The behavior was recorded for 10 min after the stimulus (Marking and Meyer 1985; Bass and Gerlai 2008) and analyzed using a tracking software such as The Real FishTracker (Buske and Gerlai 2014; Vieira et al. 2024b). Parameters such as the speed, the absolute turn angle, and the distance traveled were analyzed from the X,Y coordinates.

### Oxidative-stress related biomarkers

After the stressful event, a total of 30 animals per replica (with 5 animals per group) were collected and examined to identify any possible biochemical alterations, as reported in earlier studies by our work group (Vieira, Venancio et al. 2021, Vieira, Vieira et al. 2021a). The larvae were homogenized in a cold buffer solution containing 0.32 mM sucrose, 20 mM HEPES, 1 mM MgCl_2_, and 0.5 mM phenylmethylsulfonyl fluoride (PMSF), with a pH of 7.4 (Deng et al. 2009). This was done using the Tissuelyser II machine (30 Hz for 30 s, Qiagen, Hilden, Germany). The supernatant of the sample was obtained by spinning it at a force of 15,000 × g for a duration of 10 min using a refrigerated microcentrifuge (Labnet PrismTM R, Edison, USA) at a temperature of 4 °C. This supernatant was then used to measure several biomarkers (Felix et al. 2018; Vieira et al. 2020; Vieira, Venancio et al. 2021, Vieira et al. 2021b).

The quantification of reactive oxygen species (ROS) was conducted by employing the fluorescent probe DCFH-DA, with excitation at 485 nm and emission at 530 nm wavelengths, as described by Deng and your working group (Deng et al. 2009). The protein carbonyl (PC) was measured using the DNPH extinction coefficient at 450 nm (Mesquita et al. 2014). The amount of lipid peroxidation (LPO) was measured by quantifying the thiobarbituric acid reactive substances (TBARS) at a wavelength of 530 nm for MDA-TBA adducts and at 600 nm for non-specific reactive substances (Wallin et al. 1993).

The measurement of superoxide dismutase (SOD) activity implicated the inhibition of the photochemical reduction of nitroblue tetrazolium (NBT) at a wavelength of 560 nm (Durak et al. 1993). Catalase (CAT) activity was found at a wavelength of 240 nm by the catalytic breakdown of hydrogen peroxide, method was explained by Claiborne in 2018 (Claiborne 2018). The enzymatic activity of glutathione peroxidase (GPx) and glutathione reductase (GR) were assessed by quantifying the oxidation and reduction of NADPH at a wavelength of 340 nm (Massarsky et al. 2017). The activity of glutathione-s-transferase (GST) was assessed using the technique developed by (Habig 1981). This approach involves monitoring the conjugation of 1-chloro-2,4-dinitrobenzene (CDNB) with reduced glutathione (GSH) at a wavelength of 340 nm. The levels of reduced glutathione (GSH) and oxidized glutathione (GSSG) were modified using ortho-phthalaldehyde (OPA) and measured at an excitation wavelength of 320 nm and an emission wavelength of 420 nm (Gartaganis et al. 2007). The oxidative stress index (OSI) was determined by calculating the ratio of reduced glutathione (GSH) to oxidized glutathione (GSSG).

The activity of acetylcholinesterase (AChE) was measured using the Ellman’s approach (Ellman et al. 1961) which was adapted for use with microplates (Rodriguez-Fuentes et al. 2015). The assessment of lactate dehydrogenase (LDH) activity was performed by measuring the oxidation of NADH at a wavelength of 340 nm (Domingues et al. 2010). The quantification of total ATPase activity was conducted by assessing the quantity of inorganic phosphate (Pi) liberated, utilizing ammonium molybdate at a wavelength of 820 nm (Lanca et al. 2015).

The protein content of samples was assessed using a take 3 Multi-Volume Plate model (BioTek, Vermont, EUA) at a wavelength of 280 nm to standardize enzymatic activity. The samples were examined in duplicate using a PowerWave XS2 microplate scanning spectrophotometer (Bio-Tek Instruments, USA) or a Cary Eclipse fluorescence spectrophotometer (Varian, Palo Alto, USA) at a temperature of 30 °C and a suitable reagent blank was also included.

### Metabolic analysis

Metabolic analysis was adapted from the Reid et al. protocol (Reid et al. 2018). Resazurin stock solution of 50 mg mL^−1^ (in DMSO) was diluted to a working solution of 1 mg mL^−1^. The larvae (96 hpf, *n* = 5, 20 larvae per replica) were placed in 50-mL centrifugal tubes with E3 medium on the evening before the experiment. To these tubes, resazurin solution at 0.02 mg mL^−1^ was added; the tubes were wrapped in silver paper and then subjected to the previous stress event as described. After 1 h and 4 h, 180 µL aliquots were collected from the tubes and read by fluorescence in a microplate reader at excitation length of 530 nm and the emission length of 590 nm. Results were expressed in arbitrary fluorescence units (AFU).

### Data analysis

The Shapiro–Wilk normality test was used to test data normality, and the Brown-Forsythe test was used to test variance homogeneity prior to ANOVA. When the data had a normal distribution, the Tukey multiple comparison test was used to analyze differences between groups, and the data was reported as mean ± standard deviation. When a non-normal distribution was detected, the data was treated using the non-parametric Kruskal–Wallis analysis of variance, followed by Dunn’s test with a Bonferroni adjustment for multiple comparisons, and the data was reported as medians and interquartile ranges (25th; 75th percentiles). Also, the Student’s *t*-test (unpaired) was used to compare the metabolic changes between 1 h control, and 1 h stressed samples, and without controls at 1 and 4 h, to access if results was consequent of animal’s metabolism or solution degradation. The GraphPad Prism software (version 9) was used for the statistical analysis, and the significance level was set at *p* < 0.05.

## Results

### Cortisol, glucose, and lactate were not affected

**Fig. 1:**
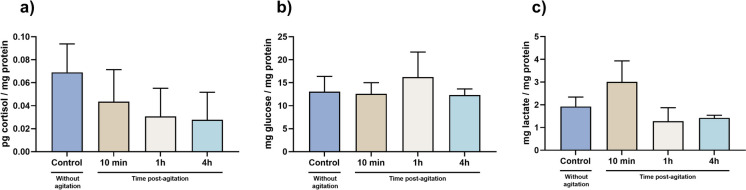
Stress parameters evaluated of control group and after 10 min, 1 h, and 4 h of stress stimulus. (**a)** Cortisol, (**b**) glucose and (**c**) lactate levels in 96 hpf zebrafish larvae. Data from at least 20 independent replicates is expressed as mean ± SD for parametric data distribution or median (25th–75th quartile) for non-parametric data. Statistical analysis was performed using one-way ANOVA followed by Tukey’s multiple-comparison test (glucose) or Kruskal–Wallis followed by Dunn’s test (cortisol and lactate)

The data for cortisol, glucose, and lactate levels are shown in Fig. 1 while raw data is shown in Table A1. No significant changes were observed for cortisol (X^2^(3) = 7.80, *p* = 0.050), glucose (F (3, 20) = 1.37, *p* = 0.289), and lactate (X^2^(3) = 7.21, *p* = 0.053) levels.

### Increased speed and distance were observed

**Fig. 2:**
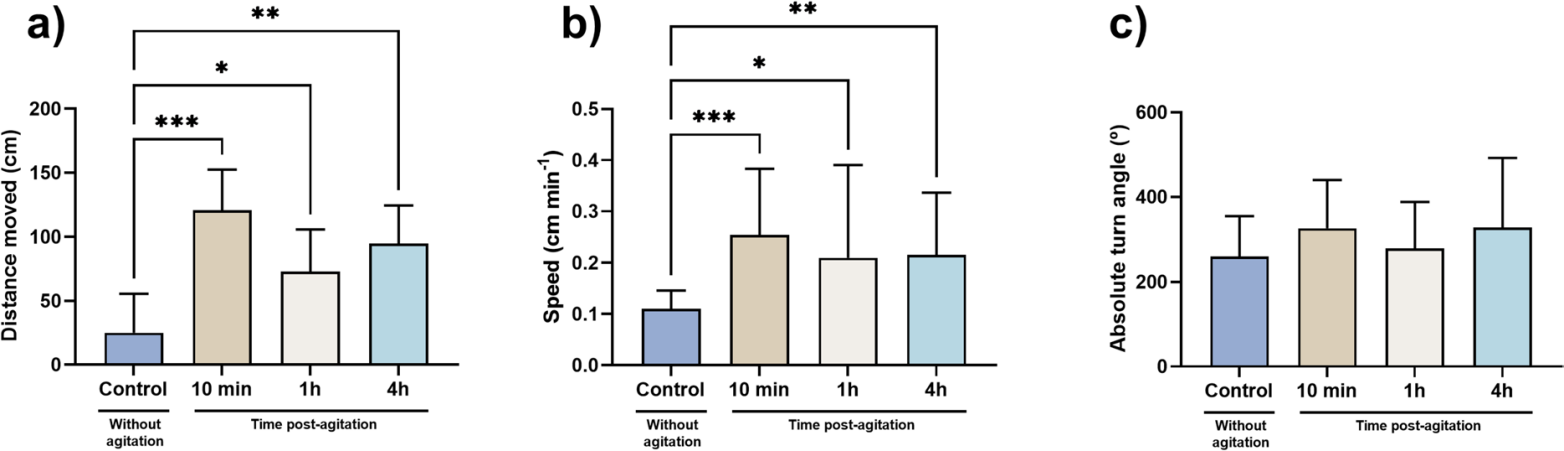
Behavior parameters, from zebrafish larvae with 96 hpf. (**a)** Distance moved, (**b**) speed and (**c**) absolute turn angle (abs turn angle). Data from at least 16 independent replicates per group is expressed as mean ± SD for parametric (abs turn angle) data distribution or median (25th–75th quartile) for non-parametric data (distance moved and speed). Statistical analysis was performed using one-way ANOVA followed by Tukey’s multiple-comparison test or Kruskal–Wallis followed by Dunn’s test. * indicates significant differences between groups (*p* < 0.05)

The results from the behavioral analysis following the stress event are presented in Fig. 2 while the original data is shown in Table A2. The distance moved (X^2^(3) = 19.28, *p* = 0.002) by animals after the stress event was significatively increased for all times evaluated when compared to control group (10 min, *p* < 0.001; 1 h, *p* = 0.024; and 4 h, *p* = 0.004). The mean speed showed a similar pattern (X^2^(3) = 18.20, *p* < 0.001), with an increase in the speed for animals subjected to the stress event in comparison to the control group (10 min, *p* = 0.001; 1 h, *p* = 0.018; and 4 h, *p* = 0.001). No significant changes were detected for the absolute turn angle.

### Significant decrease in GST and ATPase activity

**Fig. 3:**
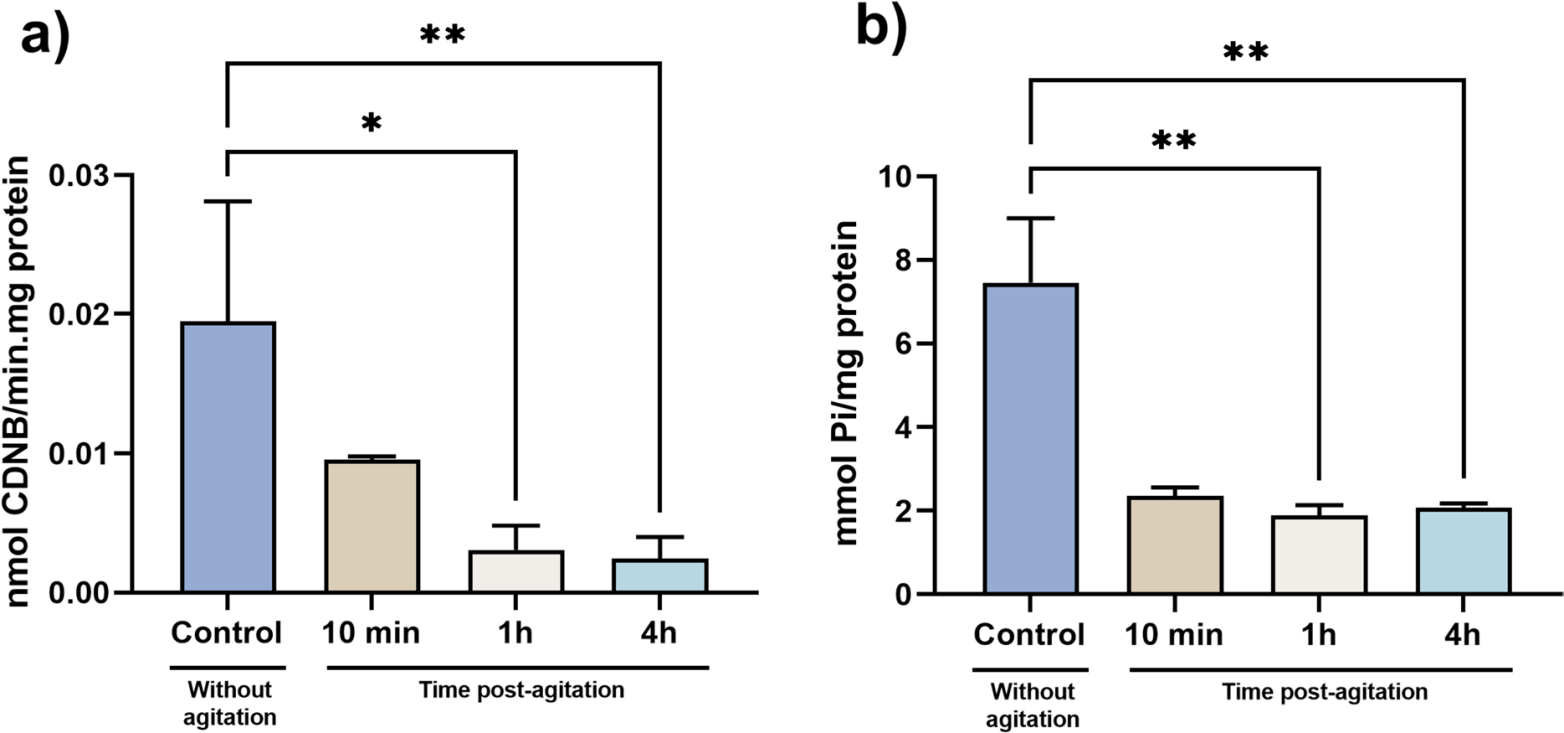
Biochemical parameters with significative differences after 10 min, 1 h, and 4 h of stress stimulus. (**a)** GST activity and (**b**) ATPase activity. Data from at least 20 independent replicates is expressed as mean ± SD for parametric data distribution or median (25th–75th quartile) for non-parametric data. Statistical analysis was performed using Kruskal–Wallis followed by Dunn’s test. * indicates significant differences between groups (*p* < 0.05)

The non-significant results obtained for the different biomarkers assessed are shown in Table 1, for ROS, SOD, CAT, GPx, GR, GSSG, OSI, LPO, PC, AChE, and LDH while the significant outcomes are shown in Fig. 3 for GSH, GST, and ATP. While a significant decrease in the GSH levels (X^2^(3) = 10.8, *p* = 0.01) was perceived from 10 min to 4 h (*p* = 0.012), no other change was depicted. When assessing GST (X^2^(3) = 14.2, *p* = 0.003) and ATPase (X^2^ (3) = 14.2, *p* = 0.003) activities, a significant decrease in both cases was noted after 1 and 4 h of the stressful event (*p* < 0.05). No other significative differences in biochemical parameters evaluated were detected.

**Table 1:** Biochemical parameters analyzed of control group without stress and after 10 min, 1 h, and 4 h of stress stimulus

	**0 min**	**10 min**	**1 h**	**4 h**	**Statistical**	***p* value**
ROS	548.1 ± 89.0	496.3 ± 82.5	495.4 ± 15.4	524.3 ± 56.3	F (3, 20) = 0.7	0.6
SOD	118.8 (86.9–186.9)	77.9 (25.3–89.6)	36.8 (25.3–89.6)	126.7 (74.9–147.1)	X^2^ (3) = 6.5	0.09
CAT	9.1 ± 7.7	13.5 ± 8.5	18.0 ± 5.2	15.8 ± 7.8	F (3, 20) = 1.3	0.3
GPx	2.5 ± 1.3	2.5 ± 0.5	1.9 ± 0.3	1.9 ± 0.4	F (3, 20) = 1.2	0.4
GR	2.7 ± 2.3	3.9 ± 2.0	5.6 ± 1.0	3.6 ± 1.6	F (3, 20) = 2.3	0.1
GSH	85.9 (83.8–100.2)^ab^	97.1 (92.8–104.0)^b^	80.1 (71.2–94.0)^ab^	80.7 (54.2–81.2)^a^	X^2^ (3) = 10.8	0.01
GSSG	75.9 ± 10.2	83.1 ± 11.0	85.2 ± 19.4	66.2 ± 17.0	F (3, 20) = 1.7	0.2
OSI	1.3 (1.1–1.3)	1.1 (1.1–1.1)	1.0 (0.9–1.1)	1.0 (1.0–1.2 s)	X^2^ (3) = 6.9	0.08
LPO	23.6 ± 6.2	29.4 ± 7.9	41.0 ± 18.7	25.7 ± 1.7	F (3, 20) = 2.5	0.1
PC	7.1 ± 1.5	6.7 ± 1.6	7.0 ± 2.1	5.5 ± 2.3	F (3, 20) = 0.7	0.6
AChE	43.5 ± 18.0	32.7 ± 9.1	26.1 ± 7.4	37.1 ± 9.3	F (3, 20) = 2.0	0.2
LDH	11.0 ± 1.7	11.9 ± 7.5	7.4 ± 0.9	8.2 ± 2.9	F (3, 20) = 1.3	0.3

Data from at least 5 independent replicates is expressed as mean ± SD for parametric data distribution or median (25th–75th quartile) for non-parametric data. Statistical analysis was performed using one-way ANOVA followed by Tukey’s multiple-comparison test (ROS, CP, LPO, CAT, GPx, GR, GSSG, AChE, and LDH) or Kruskal–Wallis followed by Dunn’s test (SOD, GSH, and OSI). Different lowercase letters indicate significant differences between groups (*p* < 0.05)

### Metabolic changes are detected up to 4 h after the stress event

**Fig. 4:**
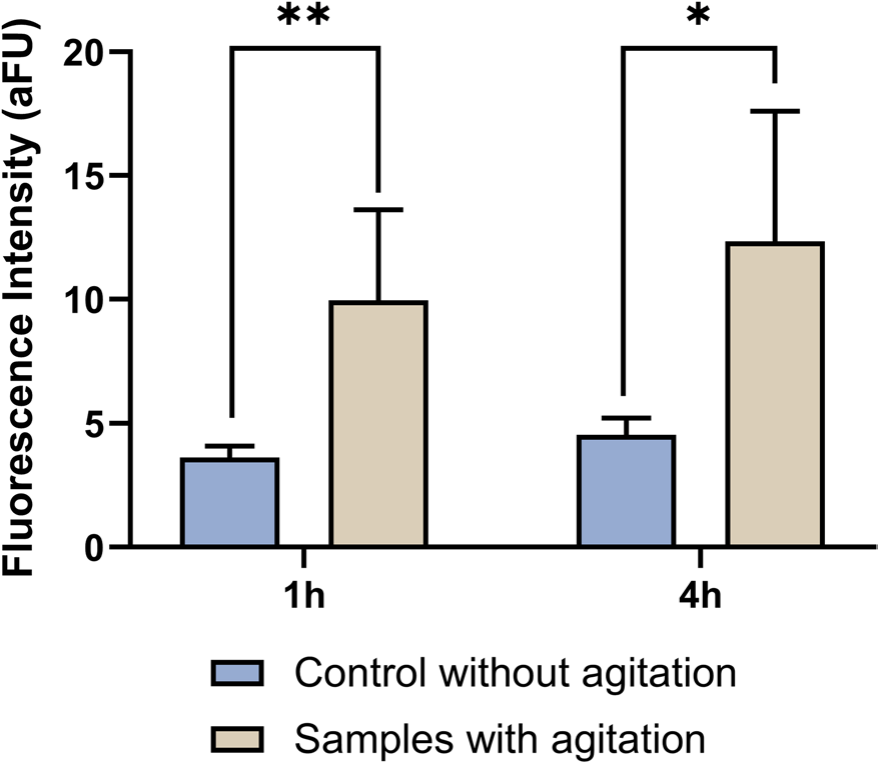
Metabolic data from 96 hpf zebrafish larvae, 1 h and 4 h after stress stimulation. Gray bars were data from group without stress and beige bars were data after stress stimulus. Data from at least 19 independent replicates, is expressed as SD parametric data. Statistical analysis was performed using *t*-test, between 1 h control animals and 1 h after the stimulus, 1 h control animals and 4 h control animals, and 4 h control animals and 4 h after the stimulus. * indicates significant differences between groups (*p < 0.05, **p < 0.005)

Figure 4 represents the metabolic analysis results, while Table A3 presents the primary data. The statistical analysis showed that there are no significant differences between control 1 h without stress and control 4 h without stress, so the color of the solution does not change, which leads us to conclude that animals not being stressed do not change their metabolic expenditure in a significant way (*p* = 0.09). When the comparison is between the non-stressed control at 1 h and the stressed animals, there is a significant increase in color intensity, which shows a greater energy consumption of the animals when they are subject to a stressful stimulus (*p* = 0.005). The deduction detained for the 4 h of testing. There was also a significant difference between the control group and the group of animals exposed to the stress event (*p* = 0.02).

## Discussion

The stress response in fish is described by the activation of the hypothalamo–pituitary–interrenal (HPI) axis (Barton 2002; Gorissen 2016; Sopinka 2016). Yet, until now, there has been no standardized and sensitive method to assess HPI axis activity, although some physical, chemical, and perceived stressors can evoke stress responses in fish Sutanto and De Kloet 1994; Campos et al. 2013; Chang et al. 2021). In addition, its relationship with behavior, metabolic rate, and biochemical changes in fish is still to be explored. Therefore, the objective of this work was to deepen the evaluation of these parameters, correlating not only the behavioral and cortisol hormonal outcomes, but also associating with other parameters related to the stress response such as lactate and glucose, as well as oxidative stress parameters, ATPase and metabolic rate. The results indicate that the levels of cortisol, glucose, and lactate remained unchanged, but there was a noticeable increase in swimming distance and speed, as well as a metabolic rate increase. A decrease in ATPase and GST activities was also detected following the vortex stress event.

Stress can be classified according to its duration as acute or chronic. An acute stress is defined as an event lasting for a period of minutes to hours, while a chronic stress usually persists from several hours to days, weeks, months, or years (Dhabhar 2013). It is mentioned by some authors that an acute or short-term stress can trigger warning responses, and after a quick action, the animal returns to the initial state (Steenbergen et al. 2011; Tort 2011, van den Bos, Cromwijk et al. 2020). The primary response to an acute stress in fish entails the release of catecholamines and the activation of the HPI axis. This activation leads to energy source mobilization (metabolic rate), reduction of glycogen stores, and an alteration in the levels of glucose along with high muscle activity, anaerobic glycolysis, alteration in lactate levels (Fazio et al. 2015), and alterations in behavior (Clark et al. 2011; de Abreu et al. 2021). Within the parameters used to evaluate the effects of stress in fishes, the levels of cortisol, lactate, and glucose are the most widely used (Ellis et al. 2012; Sopinka 2016; Sadoul and Geffroy 2019).

Cortisol is a glucocorticoid hormone released during stress events to promote mobilization of energy substrate and post-stressor metabolic recovery in teleost (Sadoul and Geffroy 2019). This hormone is generally increased in cases of stress, playing a relevant role in regulating the neuroendocrine stress response pathway and other responses as glucose pathways, energy metabolism and osmoregulation in fishes (Laiz-Carrión et al. 2002; Ellis et al. 2012; Sadoul and Geffroy 2019; Wang et al. 2022). However, in the present study, the resulting data from the cortisol evaluation showed no significant changes at 10 min, 1 h, and 4 h after the 1-min vortex stimulus. According to the literature (Lowe and Wells 1996; Stouthart et al. 1998; Alsop and Vijayan 2008; Faught and Vijayan 2022), the activation of stress-related pathways (increased cortisol and behavioral changes, modulation of aspects of intermediary metabolism, growth, and immune function) is only observed at high vortex speed. In this context, Faught and his team (Faught and Vijayan 2022) showed few variations in cortisol-related molecular levels at low rpm (250 rpm) while Castillo-Ramírez et al. (2019) and Tokarz and this team (Tokarz et al. 2013) showed an increased cortisol-related response at speeds of 330 and 600 rpm, respectively. Thus, the absence of effects at the cortisol level may be related to the low speed used during the vortex event suggesting a vortex-speed-dependent response in this parameter. Notwithstanding, cortisol is correlated with the levels of other parameters, such as glucose and lactate, which are also considered indicators of stress in fishes (Fazio et al. 2015; Sampaio and Freire 2016; Sopinka 2016). Alterations in glucose levels are generated initially by cortisol-mediated (gluconeogenesis) or by an alternative catecholamine-mediated (glycogenolysis) pathway (Dara et al. 2022), Thau, Gandhi et al. 2024). In this study, no significant alterations following the vortex event were obtained for the evaluation of glucose and lactate levels, similar to cortisol levels. Overall, these data points to the possible activation of cortisol independent pathways (Martins et al. 2012; Tokarz et al. 2013; Godoy, Rossignoli et al. 2018). In this regard, acute stress is associated with behavior alterations (Demin et al. 2021). In this study, the distance traveled by the larvae and the mean speed showed a significant increase after the vortex stress. These data agree with the fact that when there is a short-term response to stress, in the first phase, there are behavioral changes described in several species (Skomal and Mandelman 2012; Sopinka 2016; Ferrari et al. 2020; Islam et al. 2022), although in zebrafish larvae it is something relatively poorly exploited. According to the studies by Faught and his team (Faught and Vijayan 2018, Faught and Vijayan 2022), an increase of glucocorticoids mediated by corticotropin-releasing hormone (CRH) or an isolated and direct effect of CRH receptor 1 (CRHR1) is required for the initiation and maintenance of the acute stress-related behavior, which can last up to 4 h as observed in the present and other studies (De Marco et al. 2016; Faught and Vijayan 2018; Castillo-Ramírez et al. 2019). One added value of the evaluation of this parameter is that the behavior is quite sensitive, even if the levels of cortisol do not change, and can be altered and controlled by other pathways as sympathetic-adreno-medullar (catecholamines) (Joëls et al. 2008; Wong et al. 2012, ; Faught and Vijayan 2018; Das et al. 2021). Even if different pathways as sympathetic-adreno-medullar axis or hypothalamo–pituitary–interrenal (HPI) axis are activated with the stimulus, it is guaranteed that if there is some stress and stimulated pathway, this pathway can be changed (Martins et al. 2012), Godoy, Rossignoli et al. 2018). Some authors suggest this stress-mediated behavior succeeding for the interaction of catecholamines with the MR and GR receptors and has an important role in the fight or flight response to stress (Wong et al. 2012; Das et al. 2021). Also, the fact that there has been an increase in the distance traveled by the animals over time after the vortex stimulus indicates that there may have been an increased expenditure of energy in the movement of the animal, thereby increasing its metabolic rate expenditure (Priede 1985; Grantner and Taborsky 1998; Seebacher et al. 2016). The increase in metabolic rate and behavioral changes indicated that there was an energy consumption (Laiz-Carrión et al. 2002 ). The results obtained showed an increase in metabolic rate over time that can be associated with osmoregulation mechanisms to uphold internal balance at osmotic and ionic levels, ensuring proper cellular and physiological functions for their survival (Evans et al. 2005; Hwang and Lee 2007). For homeostasis to be maintained, there must be energy expenditure, which can be reflected in metabolic rate changes (Little et al. 2023). The regulation of ion and osmotic balance in teleost fish involves a complex interaction of enzymes and transporters (Laiz-Carrión et al. 2002, ). Typically, when faced with environmental fluctuations, organisms must allocate additional energy promptly to synthesize and activate the necessary enzymes, transporters, and related proteins to adapt and maintain homeostasis (Tseng and Hwang 2008). Alterations in metabolic rate, as previously mentioned, related to energy consumption can be caused by pathways such as osmoregulation (Little et al. 2023) that normally related to biochemical parameters, as the ATPase enzyme. In this study, ATPase showed a decrease in its activity over time (being more pronounced at 1 and 4 h after the vortex stimulus). According to some studies (Tseng and Hwan 2008; El Moussawi et al 2018; Little et al 2023), catecholamines (epinephrine) can alter the levels of sodium and potassium in cells, thus causing an osmotic deregulation, which in turn can give rise to a deregulation of ATPase activity and this fact can explain its decline over time. While this need to be clarified in zebrafish, a study carried out in Caco-2 cell line (El Moussawi et al. 2018) showed epinephrine to cause a decrease in ATPase activity, which is attributed to differences in the affinity of the adrenergic receptors, to their desensitization with time, or to the production of signaling intermediates that exert themselves a time dependent effect. In addition, the enzyme GST showed a decreased activity between 1 and 4 h after the vortex stimulus, although the remaining biomarkers were not altered. GST has the capacity to detoxify reactive oxygen species as a result of direct antioxidant action and has also been involved in intracellular transport and multiple products of diverse metabolic pathways (Wu et al. 2007; Monteiro et al. 2009; Oliveira et al. 2009; Zhu et al. 2011). Its levels can be influenced by adrenocorticotropic hormone (ACTH), which takes a central role in the HPI pathway, acting prior to the release of cortisol. According to (Mankowitz, Staffas et al. 1995, Stark et al. 2001), the rate of transcription of the mGSTM1 gene has a critical role in the phenotypic GST enzyme activity. Regarding zebrafish, this gene is the dominant form (Glisic et al. 2015) but not much is known about the association with GST activity although some authors have already proposed or raised this hypothesis (Donham et al. 2006; Hamed et al. 2016). Still, more studies are needed to clarify this supposition.

In summary, the gathered data suggest a vortex-speed-dependent response with activation of cortisol-independent pathways at 200 rpm, without cortisol, glucose, and lactate alterations. Moreover, this acute stress event may have activated the catecholamine pathway which is proven by the changes related to ATPase activity, metabolic rate, and increased speed and distance in behavior activity. This study has thus shown zebrafish early life stages as an innovation model that can be sensitive to an acute stimulation of agitation, reinforcing the importance of parameters more dependent on the sympathetic pathway for acute stress activities, such as the behavioral and metabolic response. Stress in early life can cause long-term changes in development, behavior, and physiology. The use of larvae at this stage complemented with the appropriate stress event offers a unique opportunity to investigate stress responses. Although further studies are required, the data obtained can be used to optimize handling and transportation practice in aquaculture improving fish welfare and enhancing the overall efficiency of fish farming operations. In addition, insights gained from this study can be potentially extrapolated to larger commercial species leading to more humane and effective aquaculture practices.

## Supplementary information

Below is the link to the electronic supplementary material.

Supplementary Material 1 (DOCX 20.5 KB)

## Data Availability

Data is provided within the manuscript or supplementary information files.
