# Supplementary material for: Behavioral, metabolic, and biochemical alterations caused by an acute stress event in a zebrafish larvae model
Source: Fish Physiol Biochem. 2024 Dec 14;51(1):25. doi: 10.1007/s10695-024-01421-7 (PMC11645430; doi:10.1007/s10695-024-01421-7)
Supplement: Supplementary file 1 — Supplementary Material 1 (DOCX 20.5 KB) [file 10695_2024_1421_MOESM1_ESM.docx]

|  | **Cortisol (pg.mg protein^-1^)** | **Glucose (mg.mg protein^-1^)** | **Lactate (mg.mg protein^-1^)** |
| --- | --- | --- | --- |
| **0 min** | 0.07 (0.05 – 0.1) | 13.0 ± 3.3 | 1.9 (1.6 – 2.3) |
| **10 min** | 0.04 (0.04 – 0.05) | 12.6 ± 2.4 | 3.0 (1.6 – 3.9) |
| **1 hour** | 0.03 (0.02 – 0.06) | 16.2 ± 5.4 | 1.3 (1.2 – 1.9) |
| **4 hours** | 0.03 (0.02 – 0.05) | 12.3 ± 0.6 | 1.4 (1.1 – 1.5) |
| **Statistical** | X^2^ (3) = 7.8 | F (3, 20) = 1.4 | X^2^ (3) = 7.2 |
| **P value** | 0.05 | 0.29 | 0.05 |

**Table A1 – Stress parameters evaluated of 96 hpf larvae, 10 minutes, 1 and 4 hours before the stress event.**

Data from at least 5 independent replicates, is expressed as mean ± SD for parametric data distribution or median (25th–75th quartile) for non-parametric data. Statistical analysis was performed using one-way ANOVA followed by Tukey's multiple-comparison test (Glucose) or Kruskal-Wallis followed by Dunn’s test (Cortisol and Lactate).

|  | **Distance moved** | **Speed** | **Abs Turn Angle** |
| --- | --- | --- | --- |
| **0 min** | 25.02 (19.34 – 55.64)^a^ | 0.11 (0.05 – 0.15)^a^ | 259.6 ± 95.25 |
| **10 min** | 120.7 (47.64 – 152.4)^b^ | 0.26 (0.18 – 0.38)^b^ | 325.9 ± 114.2 |
| **1 hour** | 73.17 (49.60 – 105.5)^b^ | 0.21 (0.15 – 0.39)^b^ | 279.8 ± 108.7 |
| **4 hours** | 94.69 (51.82 – 124.4)^b^ | 0.22 (0.18 – 0.34)^b^ | 327.6 ± 164.5 |
| **Statistical** | X^2^ (3) = 19.28 | X^2^ (3) = 18.20 | F (3, 55) = 1.12 |
| **P value** | 0.0002 | 0.0004 | 0.35 |

**Table A2 – Behaviour parameters from 96 hpf animals, exposed to stress stimulus.**

Data from at least 16 independent replicates per group, is expressed as mean ± SD for parametric (Abs Turn Angle) data distribution or median (25th–75th quartile) for non-parametric data (Distance Moved and Speed). Statistical analysis was performed using one-way ANOVA followed by Tukey's multiple-comparison test or Kruskal-Wallis followed by Dunn’s test. Different lowercase letters indicate significant differences between groups (p<0.05).

| **Hypothesis** | **n** | **Mean ±SD** | **t-value** | **p value** |
| --- | --- | --- | --- | --- |
| **1h (without stress) vs** | 5 | 3.63 ± 0.21^**^ | 3.85 | 0.005 |
| **1h (with stress)** | 5 | 9.97 ± 1.63^**^ |  |  |
|  |  |  |  |  |
| **1h (without stress) vs** | 5 | 3.63 ± 0.21 | 1.99 | 0.09 |
| **4h (without stress)** | 4 | 4.33 ± 0.30 |  |  |
|  |  |  |  |  |
| **4h (without stress) vs** | 4 | 4.33 ± 0.30^*^ | 2.98 | 0.02 |
| **4h (with stress)** | 5 | 12.34 ± 2.36^*^ |  |  |

**Table A3 –** **Metabolic data from 96hpf larvae after stress stimulation.**

Data from at least 5 and 4 independent replicates, is expressed as SD parametric data. Statistical analysis was performed using T-test, between 1hour control animals and 1 hour after the stimulus, 1 hour control animals and 4 hours control animals and 4 hours control animals and 4 hours after the stimulus. * Indicate significant differences between groups (*p<0.05, **p<0.005).
